# Prognostic Value of Metastatic Axillary Lymph Node Ratio for Chinese Breast Cancer Patients

**DOI:** 10.1371/journal.pone.0061410

**Published:** 2013-04-23

**Authors:** San-Gang Wu, Zhen-Yu He, Qun Li, Jia-Yuan Sun, Feng-Yan Li, Qin Lin, Huan-Xin Lin, Xun-Xing Guan

**Affiliations:** 1 Xiamen City Cancer Center, Department of Radiation Oncology, The First Affiliated Hospital of Xiamen University, Xiamen, People’s Republic of China; 2 State Key Laboratory of Oncology in Southern China, Department of Radiation Oncology, Sun Yat-Sen University Cancer Center, Guangzhou, People’s Republic of China; Dartmouth, United States of America

## Abstract

**Objective:**

The prevalence of breast cancer varies among countries and regions. This retrospective study investigated the prognostic value of the lymph node ratio (LNR) compared with the number of positive lymph nodes (pN) in Chinese breast cancer patients.

**Methods:**

The medical records of female breast cancer patients (N = 2591) were retrospectively evaluated. The association of LNR and TMN staging system were compared with respect to overall, disease-free, and distant metastasis-free survival.

**Results:**

Out of 2591 patients, 2495 underwent modified radical surgery and 96 received breast conserving surgery. All patients had adjuvant chemotherapy following surgery. The median follow up period 66.9 months (range 5–168 months). The 5-year and 10-year overall survival rates were 89.3% and 78.8%, respectively, and 5-year disease-free survival and distant metastasis-free survival rates were 81.6% and 83.5%, respectively. Univariate analysis indicated that in general T, pN, LNR, as well as tumor expression of the estrogen receptor, progesterone receptor, and HER2 were associated with overall, disease-free, and distant metastasis-free survival (all *P*-values <0.05). Mutlivariate analysis found pN stage and LNR were independent predictors of overall, disease-free, and distant metastasis-free survival (all *P*-values <0.001). If pN stage and LNR were both included in a multivariate analysis, LNR was still an independent prognostic factor for overall, disease-free, and distant metastasis-free survival (all *P*-values <0.001).

**Conclusion:**

Our findings support the use of LNR as a predictor of survival in Chinese patients with breast cancer, and that LNR is superior to pN stage in determining disease prognosis.

## Introduction

Metastasis to the axillary lymph nodes is a key indicator of prognosis in breast cancer. The overall 5-year survival for breast cancer patients with lymph node metastasis is 40% lower than that of patients who do not have metastasis to the lymph nodes [1], and there is almost a linear relationship with nodal disease burden and breast-cancer specific survival independent of tumor size [2]–[4]. Higher nodal disease is also associated with poor overall survival and an increased risk of locoregional recurrence [4]–[7].

Recognizing that axillary lymph node status is the most important predictor of outcomes in breast cancer, the UICC/AJCC TNM staging system for breast cancer emphasized the importance of the absolute number of positive nodes in the N classification for staging breast cancer: pN1 disease indicates 1 to 3 positive axiliary nodes, pN2 denotes 4 to 9 positive nodes, and pN3 is defined as ≥10 positive nodes [7]. Other factors that may affect the prognosis of breast cancer include the size of primary breast cancer tumor and tumor expression of the estrogen receptor, progesterone receptor, or HER2.

The potential issue of using the absolute number of affected nodes for staging is that the number of lymph nodes examined varies depending upon the surgeon’s views and technique, the patient’s anatomy, and the completeness of the pathological examination [8]. It is also not clear the minimal number of nodes that should be examined to establish lymph node metastasis. Currently, the American Joint Committee on Cancer (AJCC) recommends at least 6 auxillary lymph nodes should be removed and examined [7], [9]. In addition, the implication of the absolute number of positive auxillary lymph on staging is somewhat dependent upon the overall number of nodes examined. In other words, 3 positive nodes out of a total of 5 examined has a different implication than 3 positive nodes out of 10 examined [8]. Several studies have suggested that the ratio of involved to non-involved nodes may be an alternative, and possibly better, indicator of axillary tumor burden and consequently disease prognosis than pN staging [8], [10]–[14].

Many prior studies on the use of lymph node ration (LNR) as an indicator of breast cancer prognosis used their own LNR cutoffs making comparison of data difficult [8]. Vinh-Hung et al. [14], defined optimal cutoff points for LNRs and divided the population into those at low risk (≤0.20), moderate risk (>0.2–≤0.65) and high risk (>0.65). The LNR groups had significantly different survival rates and were more accurate than pN status for predicting survival [14]. Several other studies have supported these findings [8], [15], [16]. However, there were inconsistent findings in regard to the prognostic value of LNR on Asian breast cancer patients [17], [18].

The prevalence of breast cancer, a heterogeneous disease, varies among countries and regions. To date, few studies have investigated the prognostic value of LNR in Chinese breast cancer patients. This was a retrospective that evaluated the prognostic value of LNR compared with pN stage in breast cancer survival in a cohort of Chinese patients.

## Materials and Methods

The medical records of female patients who were treated in Sun Yat-sen University Cancer Center from January 1998 to December 2007 were reviewed and analyzed. The study was performed in accordance with the Declaration of Helsinki and was approved by the ethics committee of Sun Yat-Sen University Cancer Center. Written consent was given by the patients for their information to be stored in the hospital database and used for research.

### Study Population

Eligible patients had unilateral breast cancer with no indications of disease metastasis at diagnosis. All patients received breast-conserving surgery or mastectomy and axillary lymph node dissection. For all patients the margin of the removed tumor following surgery was negative. Neoadjuvant therapy was not performed before surgery. After surgery, all patients received at least 4 courses of adjuvant chemotherapy. Patients with >4 positive lymph nodes or who had 1–3 positive lymph nodes and had received breast-preserving surgery received postoperative radiotherapy. Depending on the TNM stage, patients who had a mastectomy were treated with radiotherapy. Patients whose tumors were positive for estrogen or progesterone receptor expression were given adjuvant endocrine therapy. None of the patients had severe comorbidities (i.e., severe heart disease, high blood pressure, rheumatic and immune diseases or a history of other cancer).

### Data Collection

The association of the risk for cancer recurrence or death with clinical and pathological factors was evaluated. These factors included age, menstrual cycle, T stage, N stage, as well as tumor estrogen receptor, progesterone receptor, and HER2 expression. The T stage and N stage were determined on the basis of AJCC TNM staging system in 2009 (7^th^ edition): N0: no lymph node metastasis; N1: metastasis of 1–3 lymph nodes; N2: metastasis of 4–9 lymph nodes; N3: metastasis of ≥10 lymph nodes.

The LNR was calculated as the ratio of metastatic axillary lymph nodes to dissected lymph nodes. The threshold of LNR was obtained using the values determined in the study of Vinh-Hung et al. [14], which used a bootstrap procedure to minimize the information loss due to grouping. They investigated the prognostic value of LNR in 1829 women with node-positive breast cancer, and the LNR threshold was defined as 0.20 and 0.65. Using this information, we classified patients into 4 LNR groups: 0, ≤0.2, 0.2 to 0.65, and >0.65.

### Study Endpoints and Follow Up

Patients were followed by clinic visit, phone, or mail at least once every 3–6 months starting one day following the surgery. Study endpoints included distant metastasis-free survival, disease-free survival, and overall survival. The survival status was obtained from medical records or by direct follow up via telephone or mail. Distant metastasis refers to the recurrence of cancer at sites distant to the breast as determined by 2 imaging examinations and, if necessary, pathological examination. Disease-free survival was defined as the length of time after treatment during which no disease was found. Death was defined at breast cancer related death.

### Statistical Analysis

Statistical analyses were performed with SPSS 15.0 Statistics Software (SPSS Inc.). Kaplan-Meier analysis with log-rank test was used to determine cumulative survival curves. Univariate and multivariate Cox proportional hazards analyses of distant metastasis-free survival, disease-free survival, and overall survival were performed to identify prognostic clinicopathologic factors for patients with invasive breast cancer. Variables which by univariate analysis had a *P*-value <0.05 were selected and evaluated by multivariate analysis. All statistical assessments were 2-sided, and statistical significance was set at *P*<0.05.

## Results

Data from 3759 patients were evaluated and 2591 met the inclusion criteria. The mean age was 46 years and most patients were premenopausal (Table 1). The majority of patients (84.0%) had T1–2 stage cancer and received modified radical surgery (96.3%). The median number of axillary lymph nodes removed was 14 and the median LNR was 0.18. About half the patients’ tumors were positive for estrogen or progesterone receptor expression and about a quarter expressed HER2 (Table 1). All patients received chemotherapy most of which included a regimen of cyclophosphamide, methotrexate, and 5-fluorouracil (CMF), or a taxane, anthracycline regimen (Table 1). Approximately one fourth of the patients had radiotherapy and over half received adjuvant endocrine therapy (Table 1).

**Table 1:** Patients’ demographics and basic characteristics (n = 2591).

Variables	n = 2591
Mean Age at diagnosis, years (SD)	46.09 (9.69)
Menopausal status	
Premenopausal	1841 (71.1%)
Postmenopausal	750 (28.9%)
T stage	
T1–2	2177 (84.0%)
T3–4	217 (8.4%)
Unknown	197 (7.6%)
LN positive	1264 (48.8%)
Median number of axillary LN dissected (range)	14 (1–73)
Median lymph node ratio (range)	0.18 (0.03–1.00)
Operation	
Modified radical surgery	2495 (96.3%)
Breast conserving surgery	96 (3.7%)
Chemotherapy	
CMF	407 (15.7%)
Taxane anthracycline-based regimen	2108 (81.4%)
Unknown	76 (2.9%)
Radiotherapy	630 (24.3%)
Adjuvant Endocrine Therapy	1760 (67.9%)
Estrogen receptor positive	1340 (51.7%)
Progesterone receptor positive	1508 (58.2%)
HER-2 positive	736 (28.4%)

CMF = cyclophosphamide, methotrexate, and 5-fluorouracil; LN = lymph node; LNR = lymph node ratio.

**Figure 1:**
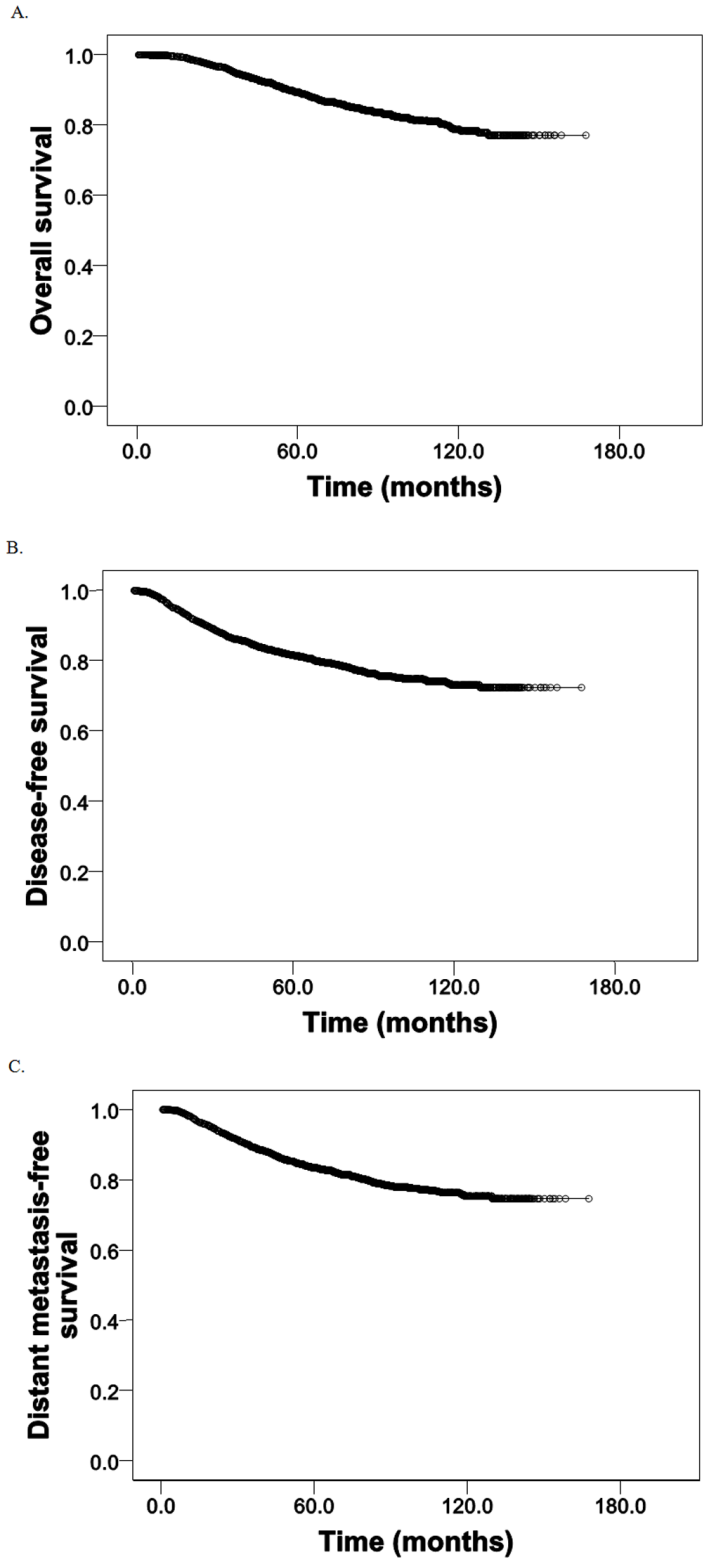
Kaplain-Meier cumulative survival curves for (A) overall survival, (B) disease-free survival, and (C) distant metastasis-free survival.

During the follow up period, 338 patients died. The median follow up time was 66.9 months (range 5 to 168 months). The 5-year and 10-year overall survival rates were 89.3% and 78.8%, respectively (Figure 1A). The 5-year disease-free survival was 81.6% (Figure 1B), and distant metastasis-free survival was 83.5% (Figure 1C).

Univariate analysis indicated that in general T and pN tumor stage, LNR, and tumor expression of estrogene, progesterone receptor or HER2, but not menopausal status or age, were potential prognostic factors for overall and disease-free survival, and distant metastasis-free survival (all *P*-values <0.05) (Table 2).

**Table 2:** The results of univariate Cox proportional hazards regression analysis of potential prognostic factors.

Characteristic	Distant metastasis-free survival		Disease-free survival		Overall survival	
	HR (95%CI)	P-value	HR (95%CI)	P-value	HR (95%CI)	P-value
Age(years)	0.99 (0.98, 1.00)	0.057	0.99 (0.98, 1.00)	0.105	1.00 (0.99, 1.01)	0.886
Menopausal status						
Post vs. Pre	1.08 (0.88, 1.32)	0.449	1.13 (0.93, 1.36)	0.211	1.20 (0.96, 1.51)	0.113
T stage						
T3–4 vs. T1–2	1.74 (1.32, 2.29)	<0.001*	1.79 (1.38, 2.31)	<0.001*	2.07 (1.53, 2.79)	<0.001*
pN stage						
N1vs. N0	1.91 (1.53, 2.38)	<0.001*	1.93 (1.57, 2.38)	<0.001*	2.04 (1.57, 2.65)	<0.001*
N2 vs. N0	2.93 (2.14, 4.00)	<0.001*	2.68 (1.98, 3.63)	<0.001*	3.05 (2.10, 4.42)	<0.001*
N3 vs. N0	6.12 (4.69, 7.97)	<0.001*	5.97 (4.64, 7.69)	<0.001*	7.00 (5.17, 9.46)	<0.001*
Lymph node ratio						
≦0.20 vs. 0	1.71 (1.34, 2.17)	<0.001*	1.72 (1.38, 2.16)	<0.001*	1.78 (1.33, 2.37)	<0.001*
0.21–0.65 vs. 0	2.93 (2.28, 3.76)	<0.001*	2.84 (2.24, 3.60)	<0.001*	3.12 (2.33, 4.19)	<0.001*
>0.65 vs. 0	6.20 (4.74, 8.12)	<0.001*	6.04 (4.67, 7.81)	<0.001*	7.06 (5.20, 9.58)	<0.001*
ER status						
Positive vs. Negative	0.62 (0.51, 0.75)	<0.001*	0.61 (0.51, 0.72)	<0.001*	0.52 (0.41, 0.64)	<0.001*
PR status						
Positive vs. Negative	0.70 (0.58, 0.85)	<0.001*	0.65 (0.54, 0.78)	<0.001*	0.54 (0.43, 0.67)	<0.001*
HER-2-neu status						
Positive vs. Negative	1.44 (1.18, 1.76)	<0.001*	1.45 (1.02, 1.76)	<0.001*	1.39 (1.10, 1.72)	0.006*

* Statistically significant.

**Figure 2:**
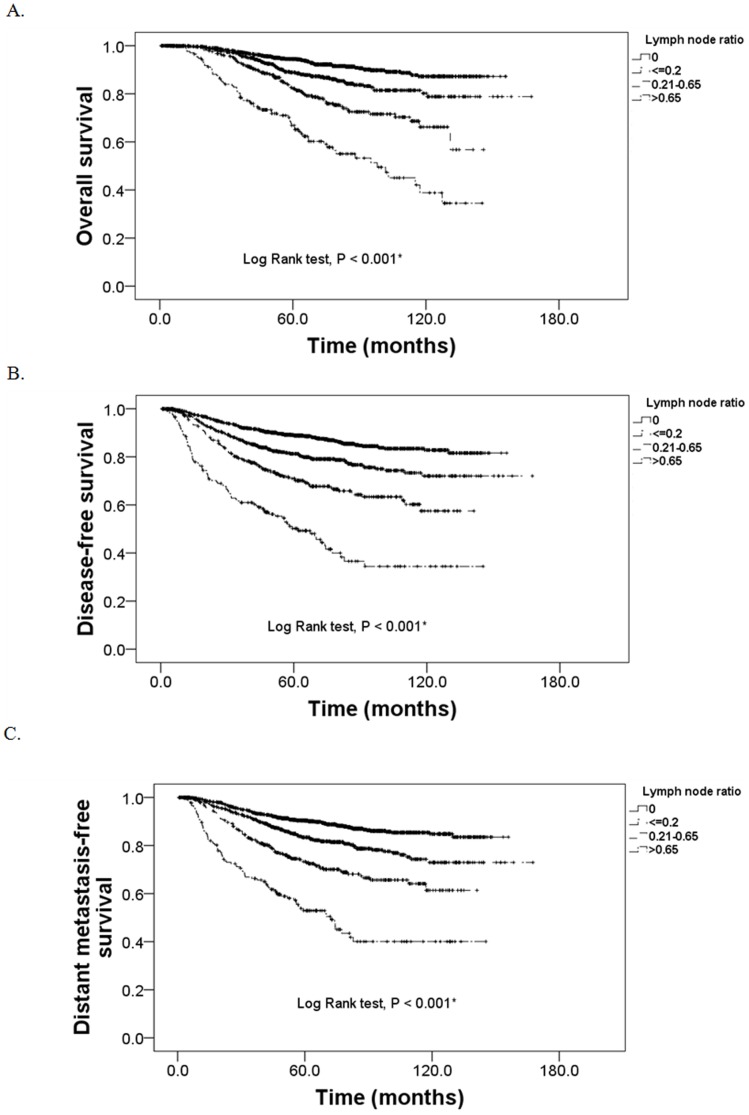
Kaplain-Meier cumulative survival curves by LNR for (A) overall survival (B) disease-free survival, and (C) distant metastasis-free survival.

Univariate analysis also indicated that patients whose LNR was ≤0.65 had significantly greater overall and disease-free survival time than those with ratios >0.65 (*P*<0.001) (Figure 2A and 2B). The proportion of patients with 5-year overall survival rates (Figure 2A) were 94.5%, 88.9%, 82.2%, and 66.8% and disease free survival rates (Figure 2B) were 89.0%, 81.3%, 71.0%, and 50.2% for patients with LNRs of 0, <0.2, 0.2 to 0.65, and >0.65, respectively. This analysis also indicated LNR ≤0.65 had significantly lower distant metastasis-free survival than those with LNR >0.65 (*P*-values <0.001) (Figure 2C). Similar to overall and disease-free survival, the lower the LNR, the greater the proportion of patients with distant metastasis-free disease (90.5%, 83.4%, 74.5%, 52.9% for LNRs of 0, <0.2, 0.2 to 0.65, and >0.65, respectively) (Figure 2C).

The univariate Cox proportional hazards analyses indicated the significant factors, T stage ER, PR and HER-2-neu, were associated with overall survival, disease-free survival, and distant metastasis-free survival (P<0.05). Multivariate analysis that adjusted for significant factors from the univariate analysis was used to assess the association of survival with LNR and pN stage either alone (model 1 or model 2) or combined together (model 3). LNR (model 2) and pN status (model 1) were associated with overall survival, disease-free survival, and distant metastasis-free survival (Table 3). Inclusion of both LNR and pN in the analysis (model 3) indicated that LNR was an independent prognostic factor for overall survival, disease-free survival, and distant metastasis-free survival (Table 3).

**Table 3:** The results of multivariate analysis of survival for lymph node ratio and pN stage.

Characteristic	Distant metastasis-free survival^1^		Disease-free survival^1^		Overall survival^1^	
	HR (95%CI)	P-value	HR (95%CI)	P-value	HR (95%CI)	P-value
***Model 1***						
pN stage						
N1vs. N0	1.83 (1.42, 2.36)	<0.001*	1.88 (1.49, 2.38)	<0.001*	2.05 (1.51, 2.79)	<0.001*
N2 vs. N0	2.82 (2.01,3.97)	<0.001*	2.59 (1.87, 3.59)	<0.001*	2.97 (1.97, 4.49)	<0.001*
N3 vs. N0	5.70 (4.24, 7.68)	<0.001*	5.42 (4.09, 7.19)	<0.001*	6.60 (4.67, 9.32)	<0.001*
***Model 2***						
Lymph node ratio						
≦0.20 vs. 0	1.68 (1.28, 2.21)	<0.001*	1.72 (1.33, 2.21)	<0.001*	1.85 (1.33, 2.58)	<0.001*
0.21–0.65 vs. 0	2.62 (1.97, 3.49)	<0.001*	2.58 (1.97, 3.38)	<0.001*	2.90 (2.05, 4.09)	<0.001*
>0.65 vs. 0	6.11 (4.52, 8.24)	<0.001*	5.77 (4.33, 7.68)	<0.001*	6.81 (4.81, 9.65)	<0.001*
***Model 3***						
pN stage						
N1vs. N0	0.91 (0.03, 31.44)	0.960	1.01 (0.04, 24.22)	0.994	1.21 (0.02, 87.69)	0.929
N2 vs. N0	1.04 (0.03, 36.64)	0.982	1.01 (0.04, 24.81)	0.993	1.27 (0.02, 94.29)	0.913
N3 vs. N0	1.40 (0.04, 49.57)	0.855	1.44 (0.06, 35.61)	0.823	1.92 (0.03, 143.31)	0.767
Lymph node ratio						
≦0.20 vs. 0	1.67 (1.27, 2.20)	<0.001*	1.71 (1.33, 2.20)	<0.001*	1.88 (1.32, 2.62)	<0.001*
0.21–0.65 vs. 0	2.63 (1.68, 3.49)	<0.001*	2.61 (2.00, 3.41)	<0.001*	3.04 (2.16, 4.28)	<0.001*
>0.65 vs. 0	6.33 (4.73, 8.47)	<0.001*	5.98 (4.53, 7.89)	<0.001*	7.18 (5.13, 10.05)	<0.001*

1 Adjusted for T stage, ER, PR and HER-.

* Statistically significant.

## Discussion

In this study, we found that breast cancer patients with lower LNR had longer overall survival, disease-free survival, and distant metastasis-free survival than patients with higher LNR values. Mutlivariate analysis found pN stage and LNR were independent predictors of overall, disease-free, and distant metastasis-free survival. If pN stage and LNR were included together in a single multivariate model, LNR was still an independent prognostic factor for overall, disease-free, and distant metastasis-free survival. These findings support the use of LNR as a prognostic factor for Chinese breast cancer patients. It also indicates that the predictive value of LNR might be superior to pN staging.

Our findings are consistent with others who have investigated the prognostic value of LNR compared to pN in breast cancer and found that the prognostic value of LNR in breast cancer is superior to that of pN stage [8], [10], [11], [14]–[16], [19], [20]. Most of these studies evaluated the relationship of LNR with survival and found that the greater the LNR the poorer the prognosis including shorter overall and disease-free survival, as well as distant metastasis-free survival time [8], [11], [15], [21]–[23]. Patients with LNR of >15% [22] or >25% [23] had a higher rate of distant-metastasis and reduced overall survival time than those with lower LNR. In one study, in univariate and multivariate analyses LNR correlated significantly with overall and disease-free survival only in a subgroup of patients who had a mastectomy and with 1–3 lymph nodes [24]. Although, our findings are consistent with these prior studies direct comparison is difficult due to difference in study design and patient populations.

LNR classification showed superiority to pN staging for the prognosis of breast cancer in current and previous studies, this superiority was also related with total number of dissected lymph nodes. Wang and his colleagues [17] reported that the superiority of LNR and pN as prognostic predictor was dependent on whether less or more than 10 lymph nodes were dissected. The median number of axillary LN dissected in this study was 14. Saxena et al. [18] reported that in combination with other factors (i.e. age, treatment, grade, tumor size and receptor status) LNR did not provide any added prognostic value for south east Asian breast cancer patients in comparison to pN except for ≥60 year old women with ER negative or grade 3 tumors. In current study, both LNR and pN status were associated with overall survival, disease-free survival, and distant metastasis-free survival in the multivariate analysis with LRN or pN separately (model 1 and model 2, Table 3). It seems LNR was not superior to pN for the prognosis of breast cancer. But, in the analysis with LNR and pN together (model 3, Table 3), LNR, but not pN, showed significant association with overall survival, disease-free survival, and distant metastasis-free survival. Our study confirmed that LNR might be better than pN for the prognosis of breast cancer.

Many of the prior studies have used diverse patient groups, and in most, the cutoffs for the nodal ratios were not determined independently or validated in alternative data sets [11]. In contrast, we used cutoffs (≤0.20, 0.2 to 0.65, and >0.65) for the categories of LNR that had previously been tested and validated via bootstrap resampling of a population-based cohort of women with lymph-positive breast cancer [20]. In addition, we evaluated a fairly homogenous population of patients with no indications of disease metastasis at diagnosis (out of 2591 patients, 2495 underwent modified radical surgery and 96 received breast conserving surgery), all of which received adjuvant chemotherapy. Our findings support the value of these cutoffs and indicate that they are applicable to Chinese breast cancer patients. The International Nodal Ratio Working Group is investigating the prognostic value of LNR in breast cancer [11], [20]. Additional studies are needed to further evaluate the use of LNR as a prognostic indicator in breast cancer.

In conclusion, our findings support the use of LNR as a predictor of survival in Chinese patients with breast cancer, and that LNR is superior to pN staging in determining disease prognosis. These findings, as well as others, indicate that cancer staging should not be confined to the TNM staging system and should at least include LNR assessment.
